# Zinc Oxide Nanoconjugates against Brain-Eating Amoebae

**DOI:** 10.3390/antibiotics11101281

**Published:** 2022-09-20

**Authors:** Ruqaiyyah Siddiqui, Anania Boghossian, Noor Akbar, Tooba Jabri, Zara Aslam, Muhammad Raza Shah, Ahmad M. Alharbi, Hasan Alfahemi, Naveed Ahmed Khan

**Affiliations:** 1 College of Arts and Sciences, American University of Sharjah, University City, Sharjah 26666, United Arab Emirates; 2 Department of Clinical Sciences, College of Medicine, University of Sharjah, Sharjah 27272, United Arab Emirates; 3 H.E.J. Research Institute of Chemistry, International Centre for Chemical and Biological Sciences, University of Karachi, Karachi 75270, Pakistan; 4 Department of Clinical Laboratory Sciences, College of Applied Medical Sciences, Taif University, Taif 21944, Saudi Arabia; 5 Department of Medical Microbiology, Faculty of Medicine, Al-Baha University, Al-Baha 65799, Saudi Arabia

**Keywords:** *Naegleria fowleri*, *Balamuthia mandrillaris*, Zinc oxide, nanotechnology, global warming, mortality, free-living amoebae

## Abstract

*Naegleria fowleri* and *Balamuthia mandrillaris* are opportunistic protists, responsible for fatal central nervous system infections such as primary amoebic meningoencephalitis (PAM) and granulomatous amoebic encephalitis (GAE) with mortality rates higher than 90%. Threatening a rise in cases is the increase in temperature due to global warming. No effective treatment is currently available. Herein, nanotechnology was used to conjugate Zinc oxide with Ampicillin, Ceftrixon, Naringin, Amphotericin B, and Quericitin, and the amoebicidal activity and host cell cytotoxicity of these resulting compounds were investigated. The compounds ZnO-CD-AMPi, ZnO-CD-CFT, ZnO-CD-Nar, ZnO-CD-AMB, and ZnO-CD-QT were found to reduce *N. fowleri* viability to 35.5%, 39.6%, 52.0%, 50.8%, 35.9%, and 69.9%, respectively, and *B. mandrillaris* viability to 40.9%, 48.2%, 51.6%, 43.8%, and 62.4%, respectively, when compared with their corresponding controls. Furthermore, the compounds reduced *N. fowleri*-mediated and *B. mandrillaris*-mediated host cell death significantly. Additionally, the compounds showed limited cytotoxicity against human cells; cell toxicity was 35.5%, 36.4%, 30.9%, 36.6%, and 35.6%, respectively, for the compounds ZnO-CD-AMPi, ZnO-CD-CFT, ZnO-CD-Nar, ZnO-CD-AMB, and ZnO-CD-QT. Furthermore, the minimum inhibitory concentrations to inhibit amoeba growth by 50% were determined for *N. fowleri* and *B. mandrillaris.* The MIC_50_ for *N. fowleri* were determined to be 69.52 µg/mL, 82.05 µg/mL, 88.16 µg/mL, 95.61 µg/mL, and 85.69 µg/mL, respectively; the MIC_50_ of the compounds for *B. mandrillaris* were determined to be 113.9 µg/mL, 102.3 µg/mL, 106.9 µg/mL, 146.4 µg/mL, and 129.6 µg/mL, respectively. Translational research to further develop therapies based on these compounds is urgently warranted, given the lack of effective therapies currently available against these devastating infections.

## 1. Introduction

*Naegleria fowleri* and *Balamuthia mandrillaris* are free-living, eukaryotic, unicellular amoebae that pose a major risk towards human health [1,2,3]. These amoebae are found across the environment globally [4]. If an individual encounters *B. mandrillaris or N. fowleri*, then infections such as primary amoebic meningoencephalitis (PAM) (by *N. fowleri*) or granulomatous amoebic encephalitis (GAE) (by *B. mandrillaris*) may occur; these infections are fatal, with mortality rates exceeding 90% [4,5,6,7,8,9,10,11,12,13,14]. The growth of these pathogens is favored in warmer temperatures; hence, the rise in temperatures due to global warming poses a serious threat, as it may promote the rise of infections [15,16,17,18,19]. Over 200 *B. mandrillaris* infection cases have been reported globally. Infected individuals have been located in the United States, Peru, South Korea, India, Japan, Thailand, China, and Australia [20,21]. Furthermore, *N. fowleri* cases have been reported globally as well. The majority of cases have been reported in the Northern USA [21]. Unfortunately, due to inadequate information and awareness, infections caused by amoebae are underreported or misdiagnosed [21]. Currently, the treatment options are limited and cause severe undesired side effects. An example of a treatment strategy currently in use is Amphotericin B; unfortunately, this drug causes nephrotoxicity. Other treatment strategies include a combinational drug therapy consisting in the administration of different antiparasitics, antibiotics, antivirals, and antifungals [16,22,23,24,25,26].

As stated earlier, amoebae are located everywhere in the environment; hence, a concept referred to as “One Health” comes into play. One health considers the interconnection between the environment, humans, and animals indicating that the health each of these elements influences that of the others [27,28].

Recently, nanotechnology has evolved and attracted the interest of the scientific community due to possibility to manipulate various physio-chemical properties of different compounds [29,30]. Specifically, nanoparticles are gaining interest due to their small size which allows their use for targeted delivery, which in turns can enhance the potency of drugs and reduce their toxicity [31]. Zinc is an essential metal element, believed to have various biochemical and physiological functions. In fact, Zinc ions are capable of causing oxidative stress and damage proteins, DNA, and lipids, inhibiting the function of respiratory enzymes and promoting free radical formation [32]. Interestingly, the use of Zinc oxide can be traced back to 2000 BC when it was used as a form of ointment to treat boils and injuries [33,34]. Moreover, Zinc oxide is greatly selective and is a semi-conductor. Researchers have tested Zinc oxide for its antibacterial properties. According to the results obtained, nano-Zinc oxide exhibited potent antibacterial effects [32,35]. In fact, a group of researchers have tested Polypyrrole–Zinc oxide against the free-living amoeba *A. castellanii*. This nanoconjugate exhibited potent amoebicidal effects against *A. castellanii* [31]. In this study, Zinc oxide nanoparticles were conjugated with ampicillin, Amphotericin B, Naringin, Quercetin, and Ceftrixon and tested against *N. fowleri* and *B. mandrillaris.* The aim of the study was to evaluate the activity of the compounds via various assays including amoebicidal, cytotoxicity and cytopathogenicity tests to determine their anti-amoebic properties.

## 2. Materials and Methods

The preparation of ZnO nanoparticles and beta-cyclodextrin-capped ZnO NPs was performed as previously described [30]. Briefly, the compounds Quercetin (QT), Naringin (NAR), and beta-Cyclodextrin (BCD) were obtained from Sigma-Aldrich, whereas the compounds Amphotericin (AMB), Ampicillin (AMPi) and Ceftriaxone (CFT) were obtained from Merck; solutions were prepared using deionized water [30]. The Zinc oxide nanoparticles were produced using the direct precipitation method, utilizing Zinc acetate dihydrate and NaOH as precursors [30].

### 2.1. Henrietta Lacks (HeLa) Cervical Cancer Cells 

Henrietta Lacks (HeLa) Cervical Cancer Cells (HeLa) cells (ATCC, CCL-2, Singapore) were acquired from the American Type Culture Collection (ATCC) to allow the conduction of various assays such as cytotoxicity and cytopathogenicity assays, as well as a feeder layer to maintain *N. fowleri* and *B. mandrillaris* cultures on [4]. These cells were grown and preserved through their cultivation in complete medium. The complete medium was the Roswell Park Memorial Medium (RPMI) containing 10% fetal bovine serum (FBS), 1% penicillin–streptomycin, 1% minimum-essential-medium amino acids, and 1% l-glutamine [4]. Furthermore, flasks containing the cells were kept at 37 °C in an incubator with 5% CO_2_ and 95% humidity [4].

### 2.2. Naegleria fowleri Culture

The HB1 ATCC 30174 strains of *N. fowleri* were obtained from the ATCC [4]. The amoebae were placed on HeLa cell monolayers which acted as a food source for the amoebae [4]. Additionally, the amoebae were maintained in a humidified incubator with 5% CO_2_ and 95% humidity at a temperature of 37 °C [7]. After 48 h on the feeder layer, the amoebae had consumed the HeLa cell monolayer; hence, they increased in number up to approximately 5 × 10^5^, of which 95% were in trophozoite form [7].

### 2.3. Balamuthia mandrillaris Culture

The ATCC 30174 strains of *B. mandrillaris* were obtained from the ATCC [4]. The amoebae were placed on HeLa cell monolayers which acted as a food source [4]. Additionally, the amoebae were maintained in a humidified incubator with 5% CO_2_ and 95% humidity at a temperature of 37 °C [7]. After 48 h on HeLa cells, the amoebae had consumed the HeLa cell monolayer; hence, they increased in number up to approximately 5 × 10^5^, of which 95% were in trophozoite form [7]. 

### 2.4. Amoebicidal Assay

We determined the antiamoebic effects of the examined compounds using amoebicidal assays [4]. In total, 2 × 10^5^ amoeba were placed in a 96-well plate, obtaining a final volume of 200 µL. Next, the amoebae were treated with the drug combinations ZnO-CD, ZnO-CD-AMPi, ZnO-CD-CFT, ZnO-CD-NAR, ZnO-CD-AMB, and ZnO-CD-QT at 100 µg/mL, after which each 96-well plate was placed in a humidified incubator with 95% humidity and 5% CO_2_ at 37 °C for 24 h.

Accurate results were ensured through the addition of positive and negative controls. Amoebae in RPMI alone served as the negative control, whereas amoebae treated with 0.25% SDS served as the positive control. Live and dead amoeba were identified through the addition of 0.1% methylene blue; the number of viable amoebae was determined by counting the living cells using a hemocytometer [4]. Additionally, compounds with significant activity were identified by conducting a Student’s t-test with a two-tailed distribution [4]. Moreover, the MIC_50_ values of the compounds ZnO-CD-AMPi, ZnO-CD-CFT, ZnO-CD-NAR, ZnO-CD-AMB, and ZnO-CD-QT were determined using concentrations of 50 µg/mL, 100 µg/mL, and 150 µg/mL [36].

### 2.5. Cytotoxicity Assay

HeLa cells were cultured in 96-well plates and treated with the compounds ZnO-CD, ZnO-CD-AMPi, ZnO-CD-CFT, ZnO-CD-NAR, ZnO-CD-AMB, and ZnO-CD-QT at 100 µg/mL. Each 96-well plate was then placed in a humidified incubator with 95% humidity and 5% CO_2_ at 37 °C for 24 h [4]. The following day, the supernatant was collected, and the toxicity of the compounds in the cells was determined through the use of a cytotoxicity detection kit. The kit measured the quantity of lactate dehydrogenase (LDH) release [37]. Accurate results were ensured by comparison to the positive and negative controls. Untreated HeLa cells represented the negative control, whereas HeLa cells treated with 1% Triton X-100 represented the positive control.

Finally, the cytotoxic properties were quantitatively determined with the formula: (absorbance of the medium from cells treated with the drugs − absorbance of the medium from negative control cells)/(absorbance of the medium from positive control cells − absorbance of the medium from negative control cells) × 100 [4]. 

### 2.6. Cytopathogenicity Assay

The amoebae-mediated host cell death was determined by treating 2 × 10^5^ amoebae with the compounds ZnO-CD, ZnO-CD-AMPi, ZnO-CD-CFT, ZnO-CD-NAR, ZnO-CD-AMB, and ZnO-CD-QT at 100 µg/mL. Each plate was then placed in the incubator for 2 h at 37 °C with 5% CO_2_ and 95% humidity. After 2 h, the treated amoebae were placed on confluent HeLa cell monolayers cultured in 96-well plates [4]. The plates containing the treated amoebae and HeLa cells were incubated for 24 h [4]. After the incubation, the supernatant was collected, and the compounds’ cytotoxicity was determined [4]. 

Accurate results were ensured by the comparison with the negative and positive controls. The amoebae on the cells served as the negative control, while the cells treated with Triton X-100 served as the positive control [4]. 

### 2.7. Statistical Analysis

The data presented show the mean ± standard error of two different independent experiments carried out in duplicates for all assays. A two-tailed distribution t-test was conducted to determine the statistical significance of the results. Additionally, the *p* values were determined to further examine and elaborate the results [4]. 

## 3. Results

### 3.1. The Conjugates of Zinc Oxide Showed Significant Amoebicidal Properties against N. fowleri and B. mandrillaris

**Figure 1:**
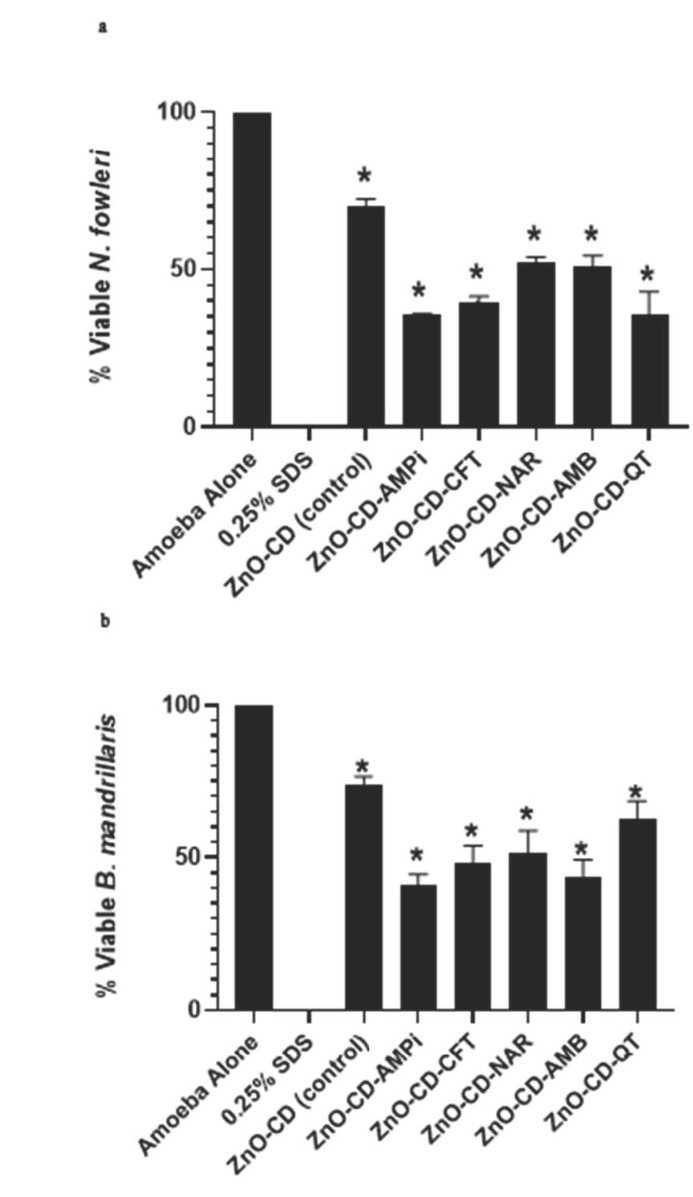
Conjugates of Zinc oxide showed significant amoebicidal properties against *N. fowleri* and *B. mandrillaris*. (**a**) At 100 µg/mL, significant amoebicidal effects of all compounds were recorded against *N. fowleri* after a 24 h incubation and (**b**) at 100 µg/mL, significant amoebicidal effects of all compounds were recorded against *B. mandrillaris* after a 24 h incubation. Data are illustrative of independent experiments and are presented as mean ± standard error. In addition, the *p*-values were determined through the conduction of a two-sample t-test, two tailed distribution; (*) is ≤ 0.05.

The amoebicidal assay allowed determining the anti-amoebic properties of the compounds against *N. fowleri* and *B. mandrillaris.* Following the incubation of the amoebae with 100 µg/mL of the compounds, significant (*t*-test, two-tail distribution, *p* ≤ 0.05) amoebicidal activity was reported. ZnO-CD-AMPi, ZnO-CD-CFT, ZnO-CD-Nar, ZnO-CD-AMB, and ZnO-CD-QT showed significant amoebicidal activity against *N. fowleri*: they reduced amoeba viability to 35.5%, 39.6%, 52.0%, 50.8%, 35.9%, and 69.9%, respectively, with respect to the negative control. In fact, the control consisting of amoebae alone exhibited 0% amoebicidal activity, whereas the positive control exhibited 100% amoebicidal activity (Figure 1). Additionally, the minimum inhibitory concentration required to inhibit 50% of parasite growth (MIC_50_) was determined to be 69.52 µg/mL, 82.05 µg/mL, 88.16 µg/mL, 95.61 µg/mL, and 85.69 µg/mL for the compounds ZnO-CD-AMPi, ZnO-CD-CFT, ZnO-CD-Nar, ZnO-CD-AMB, and ZnO-CD-QT, respectively (Table 1). The MIC_50_ of the compounds was determined through the amoebicidal assays in the presence of different concentrations of the compounds, i.e., 50 µg/mL, 100 µg/mL, and 150 µg/mL. 

Similarly, the MIC_50_ of the compounds against *B. mandrillaris* were determined to be 113.9 µg/mL, 102.3 µg/mL, 106.9 µg/mL, 146.4 µg/mL, and 129.6 µg/mL, respectively, for the compounds ZnO-CD-AMPi, ZnO-CD-CFT, ZnO-CD-Nar, ZnO-CD-AMB and ZnO-CD-QT (Table 1). Additionally, all compounds were found to exhibit significant amoebicidal activity against *B. mandrillaris.* The compounds ZnO-CD-AMPi, ZnO-CD-CFT, ZnO-CD-Nar, ZnO-CD-AMB, and ZnO-CD-QT were found to reduce *B. mandrillaris* viability to 40.9%, 48.2%, 51.6%, 43.8%, and 62.4%, respectively, with respect to the negative control. In fact, the control consisting of amoebae alone exhibited 0% amoebicidal activity, whereas the positive control exhibited 100% amoebicidal activity (Figure 1b).

### 3.2. Limited Cytotoxic Activity Was Observed against Human Cells

**Figure 2:**
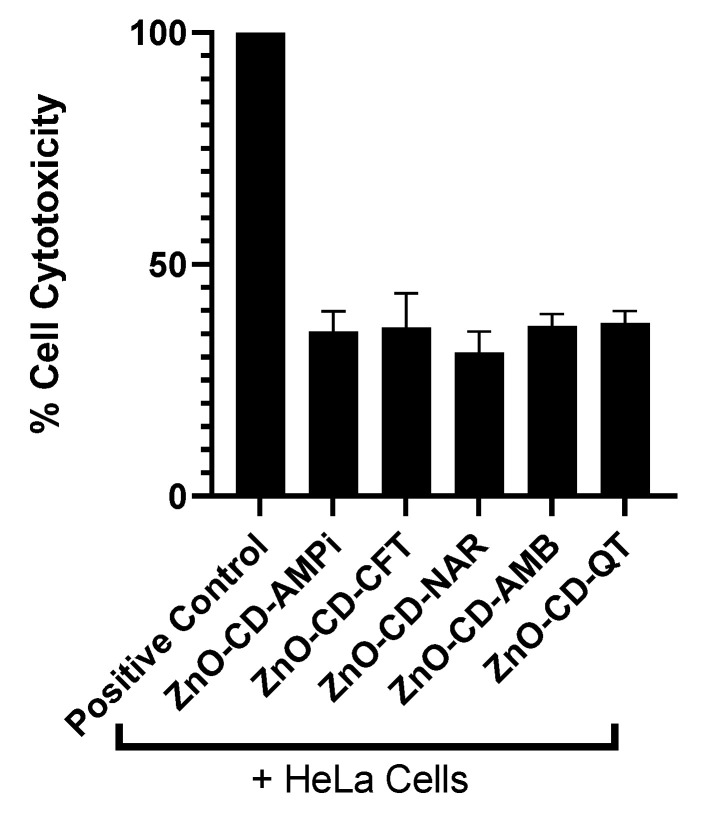
Limited cytotoxic activity was observed in human cells. Weak cytotoxic activity was determined upon the treatment of confluent monolayers of HeLa cells with the compounds at a concentration of 100 µg/mL.

To determine the toxicity of the compounds, the lactate dehydrogenase (LDH) assay was conducted using human cells. The toxicity of the compounds against human cells was determined through the LDH assay. At a concentration of 100 µg/mL the compounds ZnO-CD-AMPi, ZnO-CD-CFT, ZnO-CD-Nar, ZnO-CD-AMB, and ZnO-CD-QT showed toxicity levels of 35.5%, 36.4%, 30.9%, 36.6%, and 35.6%, respectively, compared to the positive control (Triton X-100-treated cells, which showed 100% toxicity), as reported in Figure 2. Furthermore, based on the data obtained from the LDH assay and in accordance with the ISO 10993-5, the compounds appeared to possess weak cytotoxic activity, since it was between 30.9% and 36.6% (Figure 2, Table 2). According to the International Organization for Standardization (ISO) 10993-5, if cell viability is between 60% and 80%, then a compound has limited cytotoxic activity [38,39].

### 3.3. All Compounds Promoted a Decrease in Amoeba-Mediated Cytotoxicity in Human Cells

The impact of the compounds on amoeba-mediated host cell death was determined through cytopathogenicity assays. As described earlier, the amoebae were pre-treated with the compounds, following which they were introduced to the human cells. After 24 h, the amoeba-mediated host cell death was determined, and the compounds were found to reduce amoeba-mediated cytotoxicity in human cells.

**Figure 3:**
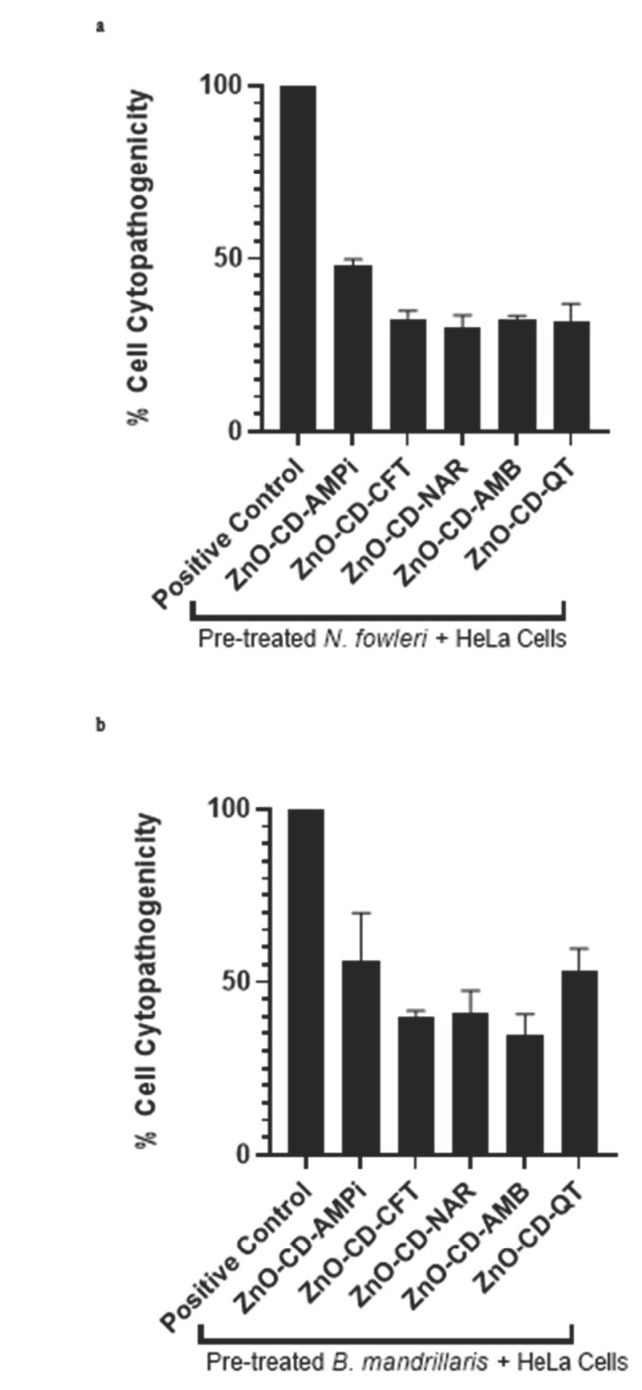
All compounds led to a decrease in amoeba-mediated cytotoxicity in human cells. (**a**) All compounds reduced *N. fowleri*-mediated cytotoxic activity and (**b**) *B. mandrillaris*-mediated cytotoxic activity in human cells. After treatment with 100 µg/mL of the compounds for 2 h, 2 × 10^5^ amoebae were placed on HeLa cell monolayers overnight, and their cytotoxicity was measured the following day.

According to the results obtained, it was found that all compounds were capable of reducing host cell death caused by the amoebae. Upon treatment of *N. fowleri* with the compounds ZnO-CD-AMPi, ZnO-CD-CFT, ZnO-CD-Nar, ZnO-CD-AMB, and ZnO-CD-QT, *N. fowleri*-mediated host cell death was found to be reduced from 100%, as measured in the negative control (amoebae alone) to 48%, 32.6%, 29.9%, 32.1%, and 31.9%, respectively (Figure 3a). Additionally, the compounds ZnO-CD-AMPi, ZnO-CD-CFT, ZnO-CD-Nar, ZnO-CD-AMB, and ZnO-CD-QT were also found to reduce *B. mandrillaris*-mediated host cell death. *B. mandrillaris*-mediated cell death was reduced from 100%, as measured in the negative control (amoebae alone), to 55.8%,39.8%, 40.8%, 34.6%, and 53.3%, respectively (Figure 3b).

## 4. Discussion

Since nanoparticles are believed to enhance drug efficiency, in previous work, various drugs were conjugated with different nanoparticles and evaluated against free-living amoebae. This method appeared efficient. In a previous study, Guanabenz was conjugated with silver and gold nanoparticles. The conjugated drug was found to possess significant amoebicidal activity against *N. fowleri* and *A. castellanii* [40]. Additionally, it was found that when Diazepam, Phenobarbitone, and Phenytoin were conjugated with silver nanoparticles, they exhibited greater effects against *A. castellanii* and *N. fowleri* compared to the drugs alone [41]. Although nanotechnology has only recently gained popularity, Zinc oxide was used as a form of treatment against boils and injuries thousands of years ago. In fact, the use of Zinc oxide dates back to 2000 BC [33]. Hence, Zinc-oxide has been evaluated against various microorganisms to determine its antimicrobial activity. For example, previous works tested drugs conjugated with Zinc oxide nanoparticles against a range of multidrug-resistant bacteria and were found to possess anti-bacterial activity [30]. Furthermore, due to the antibacterial properties Zinc-oxide nanoparticles possess, they have been considered for use in food packaging to fight foodborne diseases [42]. Additionally, Zinc oxide nanoparticles have been found to possess antifungal activity against *Botrytis cinerea* and *Penicillium expansum* [43]. Moreover, Zinc oxide nanoparticles are also believed to be able to preserve food and nutritional supplements by acting as antimicrobials [44]. 

In this study, Zinc oxide-conjugated compounds were tested for their amoebicidal properties. Through amoebicidal assays, the antiamoebic properties of the tested compounds were determined. The compounds ZnO-CD-AMPi, ZnO-CD-CFT, ZnO-CD-Nar, ZnO-CD-AMB, and ZnO-CD-QT were found to exhibit significant amoebicidal effects against both *N. fowleri* and *B. mandrillaris;* the greatest reduction in amoeba viability was achieved with the compound ZnO-CD-AMPi, which reduced *B. mandrillaris* viability to 40.9% and *N. fowleri* viability to 35.5% with respect to a negative control. The smallest anti-amoebic effects were exhibited by the conjugated compounds ZnO-CD-Nar against *N. fowleri,* with a reduction of amoeba viability to 52% with respect to the control (100% viability), and ZnO-CD-QT, which showed the smallest anti-amoebic effects against *B. mandrillaris,* reducing amoeba viability to 62.4% with respect to the control; nonetheless, the effects were significant according to the statistical analysis we conducted. Moreover, the compounds were found to reduce amoeba-mediated host cell death. This was determined through cytopathogenicity assays. It was found that ZnO-CD-AMB promoted the greatest reduction in *N. fowleri*-mediated host cell pathogenicity, whereas ZnO-CD-NAR induced the greatest reduction in *B. mandrillaris*-mediated host cell pathogenicity. Additionally, all compounds were shown to possess limited cytotoxicity when tested against human cells, as human cell viability in the presence of these compounds was greater than 60%. Moreover, the MIC_50_ of the compounds were determined. The lowest concentration necessary to inhibit *N. fowleri* growth by 50% was determined for ZnO-CD-AMPi, whereas, for *B. mandrillaris*, the lowest concentration necessary to inhibit amoeba growth by 50% was found for ZnO-CD-CFT. 

The possible mechanism of action of these compounds in *B. mandrillaris* and *N. fowleri* are not known. However, Zinc oxide nanoparticles are believed to exhibit antimicrobial activity through the alteration of the cell wall and membrane permeability by the production of reactive oxygen species in bacteria [42]. Additionally, in a study conducted on the bacterium *Campylobacter jejuni,* it was reported that a possible mechanism of action of ZnO against the bacterium is based on interactions between the compound and cell surfaces, which may be direct or electrostatic in nature. Additionally, ZnO nanoparticles may undergo cellular internalization resulting in the production of active oxygen species in the cells [45]. The compounds may also be inducing apoptosis within the amoebae, as they were previously found to induce apoptosis in human cancer cells [46]. However, this is the subject of future studies. 

The examined compounds should be tested against the cyst form of the amoebae, to determine their effects against *B. mandrillaris* and *N. fowleri* cysts. In vitro studies are necessary to determine the mechanism of action of these drugs. Next, in vivo studies need to be accomplished to understand the effects of these compounds in animal models, and translational research is urgently warranted in order to develop the much needed effective anti-amoebic drugs. These nanoconjugates could also be used as disinfectants against amoebae in water storage tanks, especially in developing countries where there is often scarcity of water; future, work is needed in this respect.

## 5. Conclusions

To conclude, Zinc oxide was conjugated with Ampicillin, Ceftrixon, Naringin, Amphotericin B, and Quericitin and was tested against amoebae to evaluate the antiamoebic properties of these conjugates. The compounds were found to exhibit significant amoebicidal properties against both *Naegleria fowleri* and *Balamuthia mandrillaris,* while exhibiting limited cytotoxic activity in human cells. Future studies will include the testing of the compounds against amoebae cysts, as well as the determination of their mechanisms of action; in vivo studies should be carried out in parallel. Moreover, the addition of these nanoconjugates to water storage tanks should be investigated.

## Figures and Tables

**Table 1:** MIC_50_ of the examined compounds against *N. fowleri* and *B. mandrillaris*. The minimum inhibitory concentration was determined for compounds against *N. fowleri* and *B. mandrillaris*. The results are expressed as percentage (%) of amoeba growth, with untreated amoeba (control) exhibiting 100% growth. Concentrations of 50 µg/mL, 100 µg/mL, and 150 µg/mL were tested against *N. fowleri* and *B. mandrillaris*.

***Naegleria fowleri***				
	**% Of Amoeba Growth**			
	**50 µg/mL**	**100 µg/mL**	**150 µg/mL**	**MIC_50_ (µg/mL)**
ZnO-CD-AMPi	65 ± 9.0	36 ± 0.4	13 ± 2.0	69.52
ZnO-CD-CFT	79 ± 6.4	40 ± 1.9	9 ± 4.5	82.05
ZnO-CD-NAR	74 ± 12.0	52 ± 2.0	14 ± 0.19	88.16
ZnO-CD-AMB	78 ± 8.3	51 ± 3.7	25 ± 0.67	95.61
ZnO-CD-QT	79 ± 10.4	36 ± 7.1	28 ± 0.51	85.69
***Balamuthia mandrillaris***				
	**% Of Amoeba Growth**			
	**50 µg/mL**	**100 µg/mL**	**150 µg/mL**	**MIC_50_ (µg/mL)**
ZnO-CD-AMPi	93 ± 3.7	53 ± 0.5	37 ± 0.7	113.9
ZnO-CD-CFT	99 ± 6.7	46 ± 4.3	27 ± 2.0	102.3
ZnO-CD-NAR	78 ± 0.9	59 ± 2.5	30 ± 1.8	106.9
ZnO-CD-AMB	86 ± 4.0	83 ± 10.4	46 ± 5.2	146.4
ZnO-CD-QT	74 ± 2.5	62 ± 6.0	43 ± 3.0	129.6

**Table 2:** Comparative graph of the amoebicidal effects and toxicity of the compounds. The compounds exhibited significant reductions in *B. mandrillaris* and *N. fowleri* viability, while maintaining limited cytotoxicity in human HeLa cells. The results presented below are expressed as percentage (%) of cell viability.

	Amoebicidal Activity against *B. mandrillaris*	Amoebicidal Activity against *N. fowleri*	Cytotoxic Activity against HeLa Cells
ZnO-CD-AMPi	35.5%	40.9%	35.5%
ZnO-CD-CFT	39.6%	48.2%	36.4%
ZnO-CD-NAR	52.0%	51.6%	30.9%
ZnO-CD-AMB	50.8%	43.8%	36.6%
ZnO-CD-QT	35.9%	62.4%	37.4%

## Data Availability

The data presented in this study are available on request from the corresponding author.
